# Enzymatic fingerprinting reveals specific xyloglucan and pectin signatures in the cell wall purified with primary plasmodesmata

**DOI:** 10.3389/fpls.2022.1020506

**Published:** 2022-10-25

**Authors:** A. Paterlini, J. Sechet, F. Immel, M. S. Grison, S. Pilard, J. Pelloux, G. Mouille, E. M. Bayer, A. Voxeur

**Affiliations:** ^1^ Laboratoire de Biogenèse Membranaire, Unité mixte de recherche (UMR5200), Université Bordeaux, Centre national de la recherche scientifique (CNRS), Villenave d’Ornon, France; ^2^ Institut Jean-Pierre Bourgin (IJPB), Université Paris-Saclay, Institut National de Recherche pour l'Agriculture, l'alimentation et l'Environnement (INRAE), AgroParisTech, Versailles, France; ^3^ Plateforme Analytique, Université de Picardie, Amiens, France; ^4^ UMRT (Unité Mixte de Recherche Transfrontaliére) INRAE (Institut National de recherche pour l'Agriculture, l'alimentation et l'Environnement) 1158 BioEcoAgro – BIOPI Biologie des Plantes et Innovation, Université de Picardie, Amiens, France

**Keywords:** plasmodesmata, cell wall, *Arabidopsis thaliana*, enzymatic fingerprinting, xyloglucans, homogalacturonans, rhamnogalacturonan I, rhamnogalacturonan II

## Abstract

Plasmodesmata (PD) pores connect neighbouring plant cells and enable direct transport across the cell wall. Understanding the molecular composition of these structures is essential to address their formation and later dynamic regulation. Here we provide a biochemical characterisation of the cell wall co-purified with primary PD of *Arabidopsis thaliana* cell cultures. To achieve this result we combined subcellular fractionation, polysaccharide analyses and enzymatic fingerprinting approaches. Relative to the rest of the cell wall, specific patterns were observed in the PD fraction. Most xyloglucans, although possibly not abundant as a group, were fucosylated. Homogalacturonans displayed short methylated stretches while rhamnogalacturonan I species were remarkably abundant. Full rhamnogalacturonan II forms, highly methyl-acetylated, were also present. We additionally showed that these domains, compared to the broad wall, are less affected by wall modifying activities during a time interval of days. Overall, the protocol and the data presented here open new opportunities for the study of wall polysaccharides associated with PD.

## Introduction

Cellular domains with specific arrays of resident macromolecules are well documented in many organisms (Scorrano et al., 2019). Plasma membrane domains of various sizes have received particular attention and have been shown to carry a plethora of functions (Gronnier et al., 2018). The domain concept can also extend to the surrounding cell wall, in biological systems where this structure is present.

Plasmodesmata (PD), membrane-lined pores across the wall of neighbouring plant cells, offer an example of this (Li et al., 2020). These conduits enable cell-cell transport and communication essential for plant growth and development (Zambryski and Crawford, 2000). PD can be regarded as membrane nanodomains in reason of their specific lipid composition (Grison et al., 2015; Liu et al., 2020) and protein population (Bayer et al., 2006; Fernandez-Calvino et al., 2011; Brault et al., 2019). In addition, their proximal wall also carries unique polysaccharide signatures (Northcote et al., 1989; Turner et al., 1994; Casero and Knox, 1995; Roy et al., 1997; Orfila and Knox, 2000; Faulkner et al., 2008).

Relevantly, the tight developmental and environmental control over the conductive status of PD is largely mediated by polysaccharides present in the local wall (Knox and Benitez-Alfonso, 2014). Callose, a polysaccharide solely composed of linear β-(1→3)-linked glucose residues, is enriched around PD (Northcote et al., 1989; Turner et al., 1994) and its amount negatively correlates with PD permeability (Amsbury et al., 2018). Callose synthase and glucanase proteins localise to PD and dynamically control the levels of this polysaccharide (Levy et al., 2007; Vatén et al., 2011). The composition of the PD wall microdomain is therefore both context dependent and differentially regulated compared to the bulk wall (via local modifying proteins). Callose might also impart mechanical properties to the PD wall domain through interactions with other wall components (Abou-Saleh et al., 2018), highlighting an additional level of regulation.

While callose has a relatively unique standing in terms of classification (Scheller and Ulvskov, 2010), cell wall polysaccharides have been traditionally divided into three groups: cellulose, hemicelluloses and pectins (Lampugnani et al., 2018). Cellulose consists of linear β-(1→4)-linked glucan chains and provides much of the mechanical strength and rigidity of plant walls (Kerstens et al., 2001). A depletion of cellulose seems to characterise PD positions in tobacco (Faulkner et al., 2008). Speculatively, a pliable wall might better accommodate dynamic changes in PD aperture or enable the modification of PD morphology itself. However, significant amounts of cellulose might actually delimit PD in onion peels (Liesche et al., 2013). Pectins, composed of homogalacturonans (HGs) and rhamnogalacturonans (RG) - I and II, and hemicelluloses such as xyloglucans, xylans, mannans and glucomannans are more heterogeneous and can present complex branched structures. As a general simplification, pectins contain large amounts of galacturonic acid residues (GalA) while hemicelluloses have β-(1→4)-linked backbones of glucose, mannose or xylose (Voragen et al., 2009; Scheller and Ulvskov, 2010). Pectin polysaccharides also display specific patterns at PD. Enrichments in (1 → 5)-α-arabinan-containing RG-I species (Orfila and Knox, 2000; Faulkner et al., 2008) and depletions of (1 → 4)-β-galactan ones (Roy et al., 1997; Orfila and Knox, 2000) have been established in tobacco, tomato and apple species. Low-esterified HGs are also abundant at PD in tomatoes and apples (Casero and Knox, 1995; Roy et al., 1997; Orfila and Knox, 2000). Pectin methylesterase (PME) proteins, which would influence this class of polysaccharides, have been localised at PD in flax and tobacco (Morvan et al., 1998; Chen et al., 2000). Their activity has been associated with the systemic spread of viruses (Dorokhov et al., 1999; Chen and Citovsky, 2003; Lionetti et al., 2014a). Overall, these examples highlight how PD wall composition might be both structurally and functionally important. The data also strongly supports the idea of PD as a spatially defined wall subdomain.

However, we still have an incomplete picture of the polysaccharide species present at PD. Most of the mentioned PD wall microenvironment data has been obtained from antibody and stain approaches. While these provide extremely valuable *in-situ* information, they are target restricted and dependent on the accessibility of the epitopes in the intact walls. We therefore considered alternative approaches to obtain broader (and more high-throughput) information at PD.

PD purification from *Arabidopsis thaliana* cell cultures has been successfully employed to produce lists of proteins closely associated with these structures (Bayer et al., 2006; Fernandez-Calvino et al., 2011; Brault et al., 2019) and to describe their lipid environment (Grison et al., 2015; Liu et al., 2020). The cells in the culture display clear PD morphologies (Bayer et al., 2004; Nicolas et al., 2017) that are likely “primary” in nature. Primary is a term used to indicate PD that have formed during cell division (Ehlers and Kollmann, 2001). The PD purification protocol involves isolation of a wall pellet (containing PD) and a subsequent mild enzymatic digestion with cell wall degrading enzymes, releasing PD-derived structures (Faulkner and Bayer, 2017). The final “PD fraction” is highly enriched in PD membrane compartments. However, the wall in the immediate proximity of PD is also likely to be co-purified. A number of extracellular cell wall modifying proteins could be indeed identified in proteomic analyses based on this approach (Knox and Benitez-Alfonso, 2014).

We reasoned that rather than treating the polysaccharides as undesired compounds co-purified with the membranous PD, the composition of the wall around PD could be studied exploiting these fractions. Xyloglucans and pectins were of particular interest as they have been speculated to be of relevance for PD function (Burch-Smith et al., 2011; Li et al., 2020; Kirk et al., 2022). In line with previous proteomic and lipidomic studies, we employed liquid cultured cells of *A. thaliana* Landberg erecta ecotype. Two ages were considered, 4 and 7 days, as changes in PD morphology had been observed between those stages and remodelling of the PD-wall environment was suggested to contribute to the process (Nicolas et al., 2017).

Combining subcellular fractionation with biochemical analyses and enzymatic fingerprinting approaches, we highlight a number of specific polysaccharide signatures in the wall fraction purified with PD. Speculations on some functional roles for the same are also provided.

## Results

### A biochemical strategy to isolate and characterise wall components in cell culture fractions

**Figure 1:**
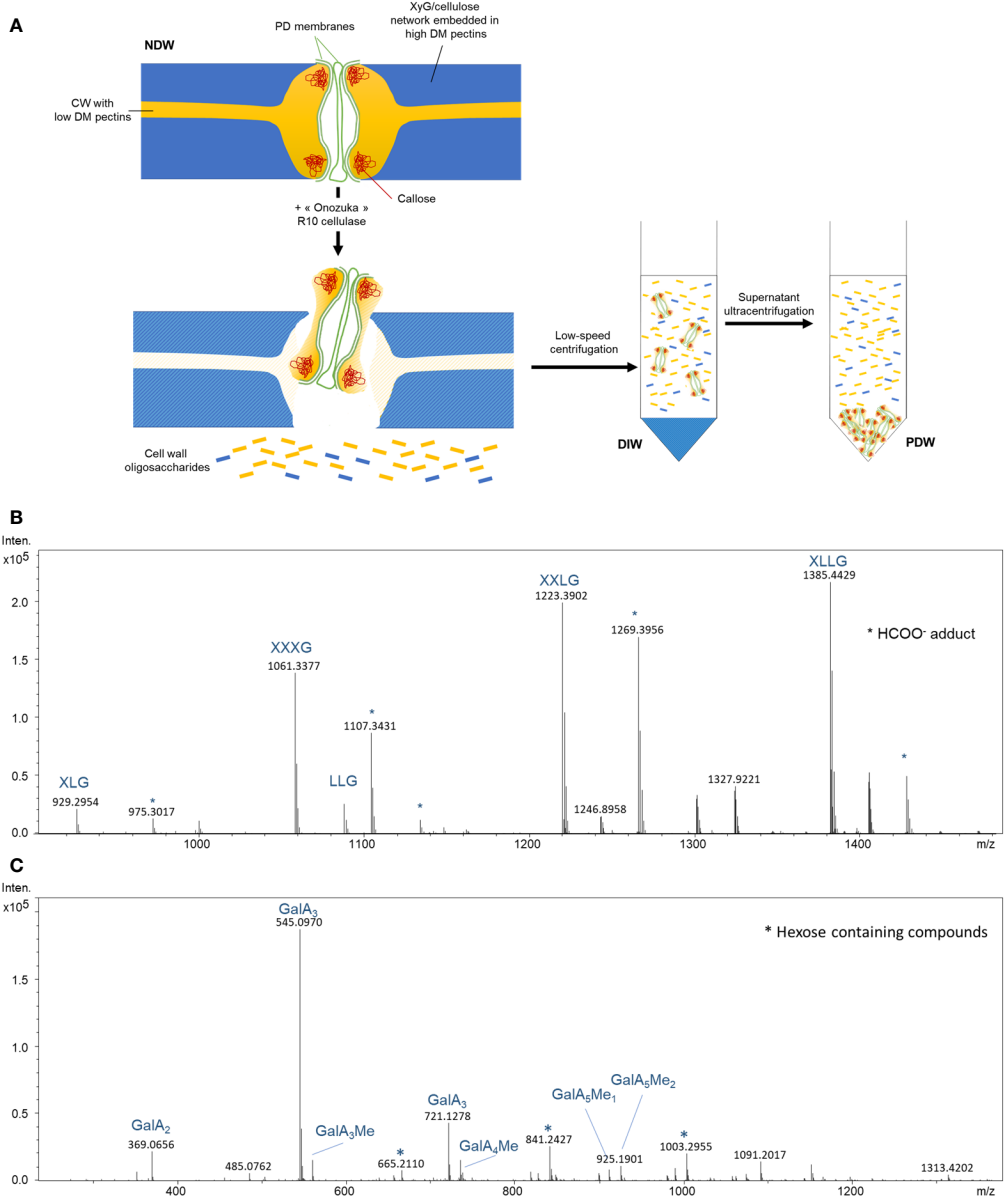
Biochemical steps involved in the release and isolation of PD from the cell wall. **(A)** Suggested model for the activity of “Onozuka” R 10 cellulase treatment on wall polysaccharides nearby PD. Subsequent key steps involved in PD purification are also depicted. DM, degree of methylesterification; XyG, Xyloglucan; NDW, non-digested wall fraction; DIW, digested wall fraction; PDW, PD wall fraction. Exposure of commercial tamarind xyloglucan **(B)** and citrus pectins **(C)** to “Onozuka” R 10 cellulase results in the production of polysaccharide fragments. MS spectrum obtained from treated samples. Intens: signal intensity; m/z: mass to charge ratio. Xyloglucan oligosaccharides follow the nomenclature described in Fry et al. (1993): G stands for glucose; X for xylose-glucose; L for galactose-xylose-glucose. OGs are named GalAxMeyAcz. Numbers indicate the degree of polymerization and the number of methyl ester groups. GalA, galacturonic acid; Me methyl-ester group. * Adduct.

The biochemical rationale for PD purification has never been explicitly discussed (Levy et al., 2007; Fernandez-Calvino et al., 2011) as it was the result of experimental trials validated by PD protein enrichments. We address the purification logic here, as it carries relevance for the specific results of this paper (**Figure 1A**).

The protocol relies on the solubilisation of a cell wall domain, that of PD, *via* a mild treatment with a commercial cellulase mixture (Cellulase “Onozuka” R-10 from *Trichoderma viride*). The mixture contains hemicellulase and pectinase activities in addition to the titular cellulase one (Beldman et al., 1985). The adjective mild is used in comparison to protoplasting approaches, which aim to fully digest the cell wall and usually employ additional enzymes (Pasternak et al., 2021). A shorter exposure and a lower concentration of enzyme (per amount of substrate) are employed during PD purification. These differences relate to the aim of solubilising a wall domain, rather than specific polysaccharides. The latter endeavour would require chelators and other specific substances, which are not employed. The cell wall digestion is also intentionally partial as a longer digestion could solubilise most of the cell wall.

The assumption would be that the “Onozuka” R-10 mixture digests cellulose, releasing PD structures. Secondary enzymatic activities in the mixture (in addition to cellulase) have received less attention by the PD community. However, by treating pure commercial xyloglucans and pectins with the “Onozuka” R10 mixture, a number of polysaccharide fragments and by-products became visible upon High Pressure Size Exclusion chromatography (HPSEC) combined with high resolution mass spectrometry (HRMS) (**Figures 1B** **, C**; **Supplementary Figures S1A, B**). Our analysis shows that the mixture can actually easily degrade low methyl-esterified pectins in di-, tri- and tetrameres of GalA. PD are embedded in low-esterified HGs wall domains (Roy et al., 1997; Orfila and Knox, 2000) so, in our view, processing of these highly digestible components is more likely to explain the release of PD structures than cellulose digestion (**Figure 1A**).

Once the attachment sites are partially digested, PD (membranes and associated wall components) can be released from the rest of the wall. These small suspended domains containing PD are then separated from the bulk wall by a low speed centrifugation (**Figure 1A**). To then isolate PD from soluble digestion products (oligosaccharides released by the “Onozuka” R-10 enzymatic mixture), an ultracentrifugation step is performed. PD are expected to be the biggest product because of the presence of callose - which would not be digested by this enzymatic mixture - and of retained membranous components. These structures can therefore be easily separated from other degradation products (**Figure 1A**)

The standard PD purification strategy described in Faulkner and Bayer (2017) and applied in a number of papers in the field (Fernandez-Calvino et al., 2011; Brault et al., 2019) was re-deployed in this study just changing the fractions being collected. Cells were passed through a N_2_ disruptor device and the wall pellet of the lysed cells was collected. It was defined as the non-digested wall (NDW) fraction. The pellet (bulk wall) after mild “Onozuka” R-10 cellulase enzymatic digestion was defined as the digested wall fraction (DIW) and the pellet obtained from the supernatant after ultracentrifugation as the PD wall (PDW) fraction (**Figure 1A**).

**Figure 2:**
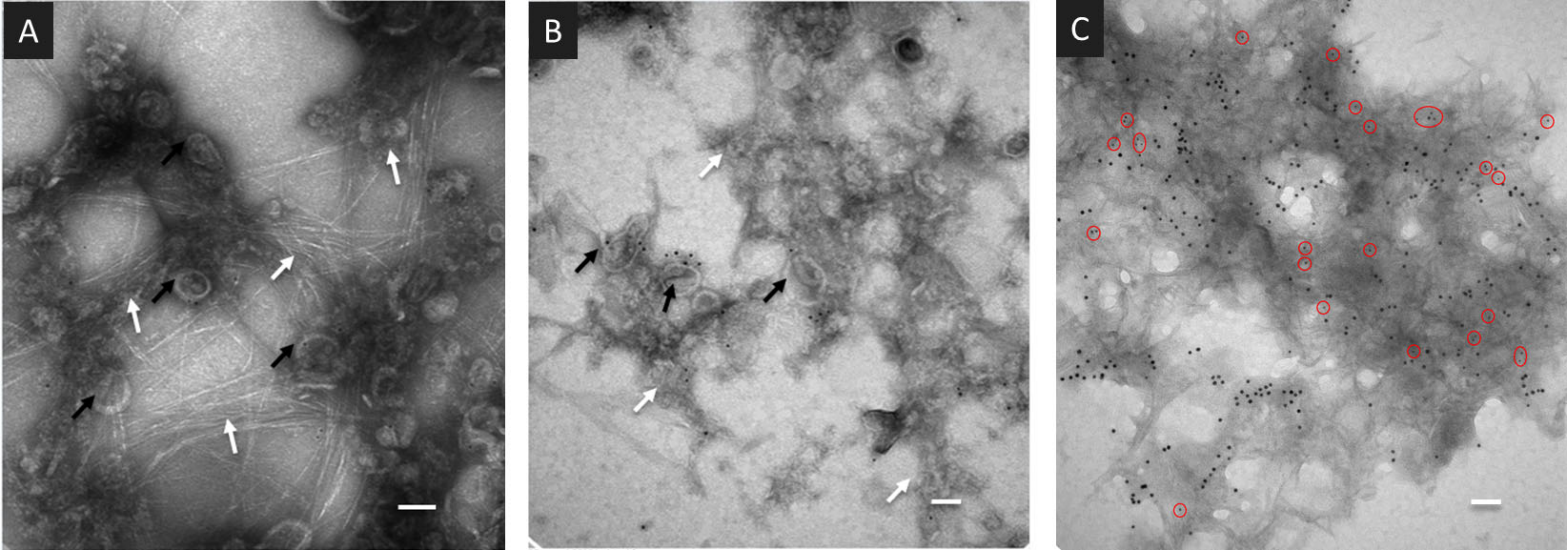
Wall-derived material is present in PDW fractions purified from *A.thaliana* cell cultures. **(A, B)** TEM images of the PDW fractions containing PD-derived vesicles (black arrows) labelled with antibodies against the PDLP1 protein and colloidal gold particles. Fibrillar **(A)** and anamorphous **(B)** structures are visible (white arrows). **(C)** TEM images of the PDW fraction labelled with antibodies against the PDCB1 protein (15nm - larger - colloidal gold particles) and callose (5 nm - smaller - gold particles highlighted by red circles). Panels **(A, B)** have scale bars of 50 nm; panel **(C)** has a scale bar of 200 nm.

Transmission electron microscopy (TEM) observations provided early indications that cell wall polysaccharides are indeed retained during PD purification (Fernandez-Calvino et al., 2011). Microfibril structures are visible among fixed PD derived vesicles labelled with antibodies against Plasmodesmata Located Protein 1 (PDLP1) (Thomas et al., 2008) and colloidal gold particles (**Figures 2A, B**). A range of polysaccharides, among which callose and cellulose (Cifuentes et al., 2010; Ding et al., 2014) has this specific appearance. The presence of callose in PDW was directly confirmed by immunolabeling with antibodies against Plasmodesmata callose binding proteins (PDCB1) (Simpson et al., 2009) and callose itself, combined with colloidal gold (**Figure 2C**). While the extracellular domains of PDLPs’ display structural homology to fungal lectins, capable of binding a range of carbohydrates (Vaattovaara et al., 2019), the extracellular domains of PDCBs specifically recognise callose (Barral et al., 2005; Simpson et al., 2009). A reduction in the number of microfibril structures can be observed comparing PDW to DIW fractions (**Supplementary Figure S2**). Extensive anamorphous-looking material is also visible (**Figures 2B** **, C**) and might correspond to non-crystalline hemicelluloses and/or pectins, presenting less defined appearances under TEM (Chen et al., 2016; Wathoni et al., 2019).

**Figure 3:**
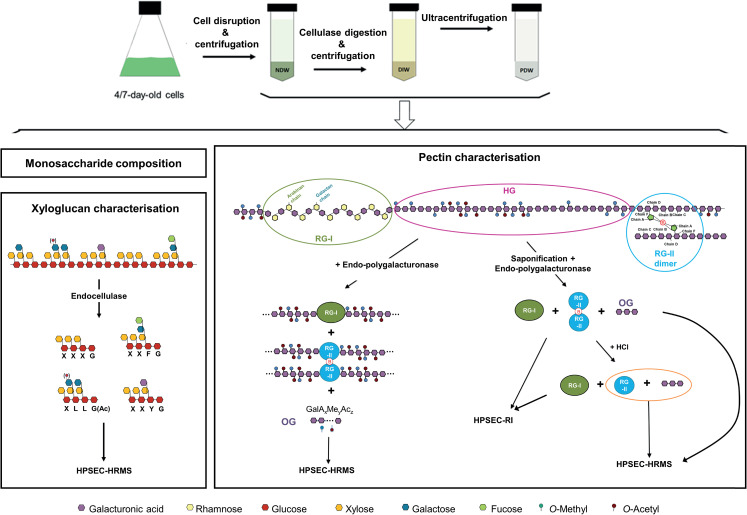
Purification steps required to obtain cell wall fractions and biochemical analyses to identify the polysaccharides present in the same. NDW, Non digested cell wall; DIW, Digested cell wall; PDW, plasmodesmata cell wall; RG-I, Rhamnogalacturonan I; RG-II, Rhamnogalacturonan II; HG, Homogalacturonans; HPSEC, High-pressure size exclusion chromatography; HRMS, High resolution mass spectrometry; RI, refractive index; OG, Oligogalacturonides; Ac, Acetyl-ester group; Me, Methyl-ester group; OGs are named GalAxMeyAcz. Numbers indicate the degree of polymerisation (DP) and the number of methyl- and acetyl-ester groups, respectively.

The NDW, DIW and PDW fractions were subjected to the analyses depicted in the lower part of **Figure 3**. We performed a monosaccharide analysis (Fang et al., 2016) to obtain broad information on the cell wall sugars (**Figure 3**). Specific structural features were then defined using enzymatic fingerprinting approaches. By digesting cell walls with enzymes possessing well-defined cleavage specificities, a unique fingerprint of oligosaccharides can be produced. The subsequent detailed analysis of these products by HPSEC-HRMS (Voxeur et al., 2019) gives structural information on the original polysaccharide (Limberg et al., 2000). The validity of the approach has been previously confirmed using immunolabeling approaches (Jobert et al., 2022 - bioRxiv; Cavalier et al., 2008)

An endo-cellulase treatment, which cuts the xyloglucan backbone after non-substituted glucose residues (**Figure 3**; Lerouxel et al., 2002), was employed to describe xyloglucan structures. The xyloglucan oligosaccharides produced are characterised by their monosaccharide sequence, their branching and their acetylation status (Fry et al., 1993).

Digestion with endo-polygalacturonase (cutting between two non-methyl-esterified galacturonic acids of HGs) (**Figure 3**; Pedrolli et al., 2009) combined with oligosaccharide analysis revealed the structure of oligogalacturonan (OG) stretches derived from the digestible HG backbone. OGs are linear and are characterised by their degree of polymerisation (DP) and their acetylation (Ac) and methylation status (Me). They are named GalAxMeyAcz where the numbers indicate the degree of polymerization and the number of methyl- and acetyl-ester groups, respectively (Voragen et al., 2009). A similar analysis, was performed after saponification which largely strips pectins of their methyl- and acetyl-ester chemical modifications (Séveno et al., 2009) and creates a fully digestible HG backbone. Digestion with endo-polygalacturonase of these saponified pectins results in the release of OGs, RG-I and RG-II dimers, which can be next separated by size-exclusion chromatography and detected by refractive index (RI) (**Figure 3**; Ishii et al., 1999; O'Neill et al., 2001). Since RG-II dimers, unlike monomers, cannot be detected by mass spectrometry, we performed a hydrochloric acid (HCl) treatment, which releases RG-II monomers from boron cross-linking (O'Neill et al., 1996). RG-II monomer and OGs were then analysed by HPSEC-HRMS (**Figure 3**).

### “Onozuka” R10 cellulase treatment solubilizes fucosylated and galacturonic acid-containing xyloglucans, HG, RG-I and RG-II from the wall

As discussed, treatment with a commercial cellulase mixture is necessary to release PD from the rest of the wall (**Figures 1**, **3**; Faulkner and Bayer, 2017). Polysaccharides lost from the NDW fraction will therefore include those present in the proximity of PD (PDW fraction) plus those directly processed by the enzymes. A detailed comparison between NDW and DIW fractions was performed to better define which polysaccharides are lost upon “Onozuka” R10 cellulase treatment.

In the monosaccharide analysis we focused on fucose, galactose, xylose, mannose, rhamnose, arabinose, glucose and galacturonic acid residues, which would be derived from hemicelluloses and pectin components (Voragen et al., 2009; Scheller and Ulvskov, 2010). No robust changes in the levels of any of these sugars were observed upon “Onozuka” R10 cellulase digestion, in both fractions derived from 4- or 7- day-old cell cultures (**Supplementary Figure S3**). This suggests that cellulose, pectins and hemicelluloses are roughly equally digested upon the PD purification process.

**Figure 4:**
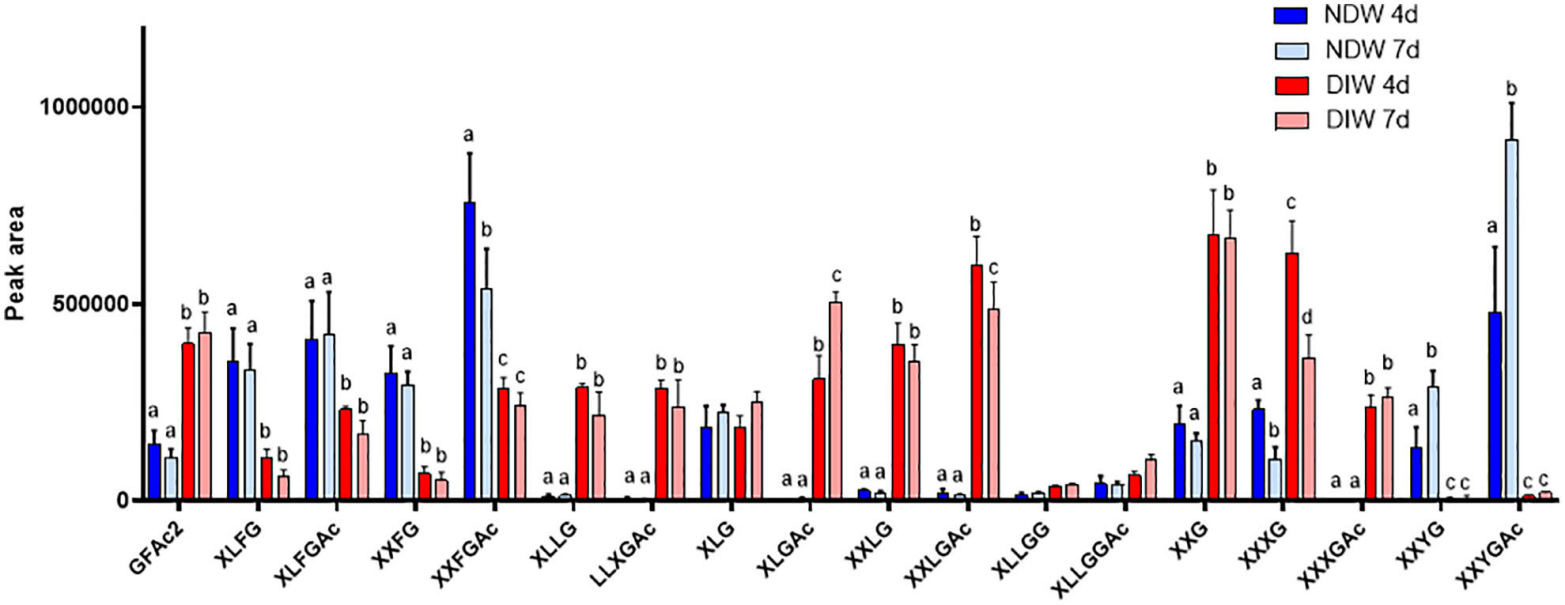
Enzymatic fingerprinting of xyloglucans in NDW and DIW fractions. Histograms display the peak area of each xyloglucan oligosaccharide released upon endo-cellulase digestion of NDW and DIW fractions obtained from 4- and 7-day-old *A.thaliana* cell cultures. Data are the mean of three biological replicates, error bars are the standard deviation. Lowercase letters indicate significant differences in the amounts of specific xyloglucans between fractions (two-way ANOVA, p < 0.05). Oligosaccharide nomenclature follows that described in Fry et al. (1993): G stands for glucose; X for xylose-glucose; L for galactose-xylose-glucose; F for fucose-galactose-xylose-glucose; Y for galacturonic acid-xylose-glucose; Ac: acetyl ester group.

Upon xyloglucan fingerprinting, we observed a significant loss of fucosylated and galacturonic acid-containing xyloglucans (such as XXFG and XXYG) (**Figure 4**) in DIW fractions. This loss, together with cellulose degradation, causes increases in the abundance of other xyloglucan species (e.g. the galactose containing XLLG and XXLG forms) (**Figure 4**), as individual peak areas are scaled by the alcohol insoluble residue (AIR) weight.

**Figure 5:**
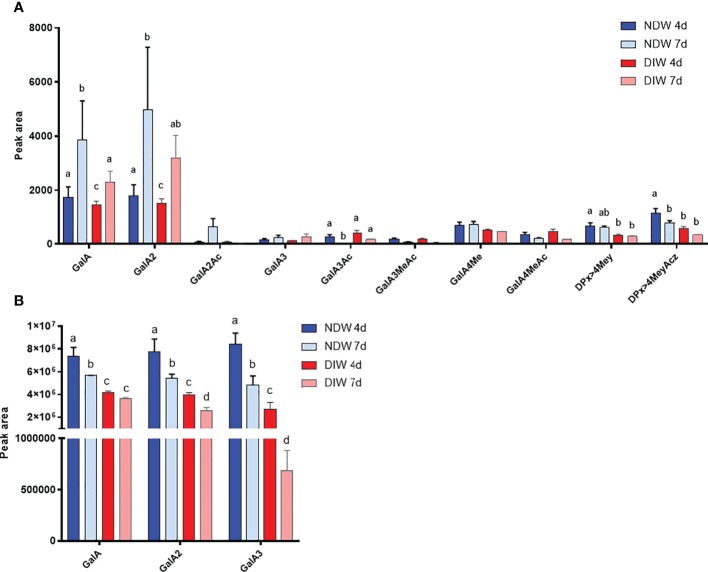
Enzymatic fingerprinting of HG in NDW and DIW fractions. Histograms display the peak area of each OGs analysed by HPSEC-HRMS and released upon endo-polygalacturonase digestion of non-saponified **(A)** and saponified **(B)** NDW and DIW fractions obtained from 4- and 7-day-old *A thaliana* cell cultures. Data are the mean of three biological replicates, error bars are the standard deviation. Lowercase letters indicate significant differences in the amounts of specific OGs between fractions (two-way ANOVA, p < 0.05). OGs are named GalAxMeyAcz. Numbers indicate the degree of polymerization and the number of methyl/acetyl ester groups. GalA, galacturonic acid; Me: methyl-ester group; Ac, acetyl-ester group; DP, degree of polymerisation. OGs larger than GalA4 are grouped together. z and y represent potential higher levels of acetylation/methylation in those longer OGs.

HG fingerprinting also revealed that methyl-esterified pectins were released upon “Onozuka” R10 cellulase digestion. The NDW and DIW enzymatic fingerprinting highlighted solubilisation of HG containing monomeric galacturonic acid, GalA2 and methyl-esterified stretches (DPx>4Mey and DPx>4MeyAcz) (**Figure 5A**) upon PD extraction. A full profile of the OGs detected in NDW and DIW is provided in **Supplementary Figure S4**. As HG methyl-esterification prevents endo-polygalacturonase digestion (Séveno et al., 2009), we performed saponification on the fractions prior to digestion in order to assess the total amount of pectins released upon PD extraction. An increase of GalA3 was observed in fractions from both 4- and 7-day-old cultures. This suggests that GalA3 is likely derived from non and/or lowly methyl-esterified wall regions, obtained by saponification. The amount of this OG is indeed negligible before treatment (**Figures 5A** **, B**). GalA/GalA2, conversely, are more likely to correspond to short de-methyl-esterified stretches present among highly methyl-esterified ones prior to saponification (**Figure 5A**). This analysis confirmed that methyl-esterified pectins were released upon the PD purification process.

Saponification (when combined with endo-polygalacturonase digestion) additionally allows pure RG-II and RG-I isolation (Sun et al., 2019). RG-II is a complex polysaccharide with multiple different side branches. Six conserved types (**Supplementary Figure S5**) have been described to date (O'Neill et al., 2004; Ndeh et al., 2017).

**Figure 6:**
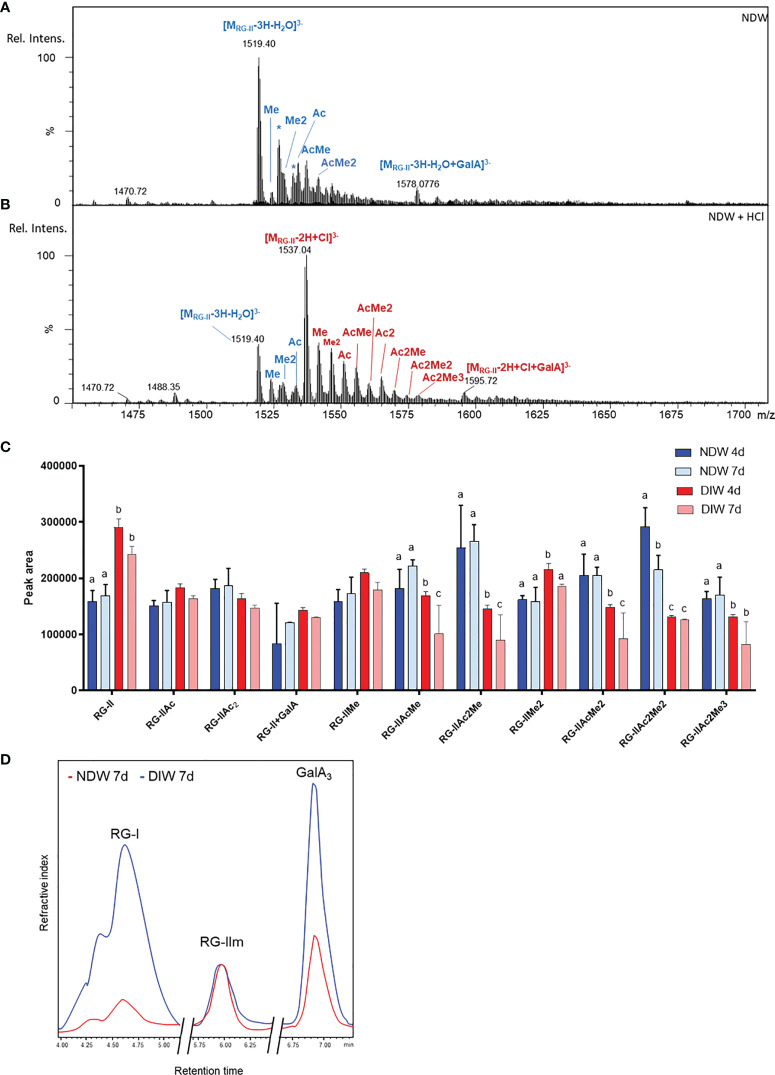
Rhamnogalacturonan analysis of NDW and DIW fractions. Mass spectrum of complete RG-II forms in NDW fractions derived from 7-day-old *A.thaliana* cell cultures before **(A)** and after **(B)** HCl treatment. Chloride adducts are represented in red and dehydrated forms in blue. Asterisks correspond to Na adducts. RG-II: rhamnogalacturonan-II; GalA; galacturonic acid; Rel. Intens.: relative signal intensity; m/z: mass-to-charge ratio; Me and Ac denote methylation and acetylation in RG-II species. **(C)** Histograms displaying the peak area of each RG-II form released upon endo-polygalacturonase digestion of saponified and HCl-treated NDW and DIW fractions obtained from 4- and 7-day-old *A thaliana* cell cultures. Data are the mean of three biological replicates, error bars are the standard deviation. Lowercase letters indicate significant differences in the amounts of specific RG-II species between fractions (two-way ANOVA, p < 0.05). **(D)** HPSEC-RI quantification of RG-I, RG-II monomers and GalA3 in HCl-treated NDW and DIW fractions derived from 7-day-old *A.thaliana* cell cultures. The x axis is broken.

In the HPSEC-HRMS analysis of saponified and PG-digested wall fractions we observed a main three-charged ion at monoisotopic m/z 1518.7 (**Figure 6A** - NDW as an example) of which the retention time matched those obtained for RG-II monomers (**Supplementary Figure S5**). Since RG-II is a high molecular weight molecule, the most abundant species in the pattern was actually the di-isotopic one, containing two ^13^C atoms (m/z 1519.4; **Figure 6A**). Specifically, the ion corresponds to a molecular formula of C_166_H_246_O_145_, fitting a dehydrated RG-II backbone containing 8 GalA substituted with the highly conserved side chains A, C and D, a B side chain hexasaccharide and two distinct α-L-arabinofuranose substituents (E and F; **Supplementary Figure S5**). We attributed other ions to mono or di-methyl-etherified GalA (m/z 1523.3 and 1528.0) and mono-acetylated side chain A (m/z 1532.7) (**Figure 6A**). A complete RG-II form is therefore present in the walls of *A. thaliana* cell cultures. After HCl treatment, which releases the RG-II monomers from boron cross-linking (O'Neill et al., 1996; **Supplementary Figure S6**), the main ions were observed as chloride adducts and more acetylated RG-IIs were detected in the fractions (**Figure 6B** - using NDW as an example). RG-II dimers have been reported to be more acetylated than monomers (O’Neill et al., 2020) (**Figure 6** **B**).

Most of these RG-II pectins remained in the DIW fraction after “Onozuka” R10 cellulase digestion (**Figure 6C**). Only a specific population of methyl-acetylated forms was solubilized (**Figure 6C**). As individual peak areas are scaled by the AIR weight, this results in the increase of RG-II content relative to other RG-II species in DIW (**Figure 6C**).

HPSEC-RI on wall material derived from 7-day-old cell cultures also confirmed that the amount of RG-II is largely unaltered between NDW and DIW fractions (**Figure 6D**). The loss of low methyl-esterified pectins (GalA3) observed in **Figure 5** **B** was similarly supported. HPSEC-RI additionally displayed a significant loss of RG-I-containing pectins upon “Onozuka” R10 cellulase digestion (**Figure 6D**).

Overall, this cellulase digestion treatment seems to result in loss of fucosylated and galacturonic acid-containing xyloglucans; methyl-esterified HG; RG-I; and methyl-acetylated RG-II forms from the wall of *A. thaliana* cells.

### The PDW fraction presents fucosylated xyloglucans, low methyl-esterified HGs, RG-I with limited branching and acetylated RG-II

To determine which of the solubilised polysaccharides belonged to the proximal PD wall and which were the result of direct enzymatic activities, we analysed PDW fractions. PDW pellets are obtained from purification of polysaccharides released upon “Onozuka” R10 cellulase treatment (**Figures 1**, **3**; Faulkner and Bayer, 2017). Comparing these fractions with NDW ones (representative of the general wall of plant cell cultures) enables us to determine the presence of unique polysaccharide signatures at PD.

**Figure 7:**
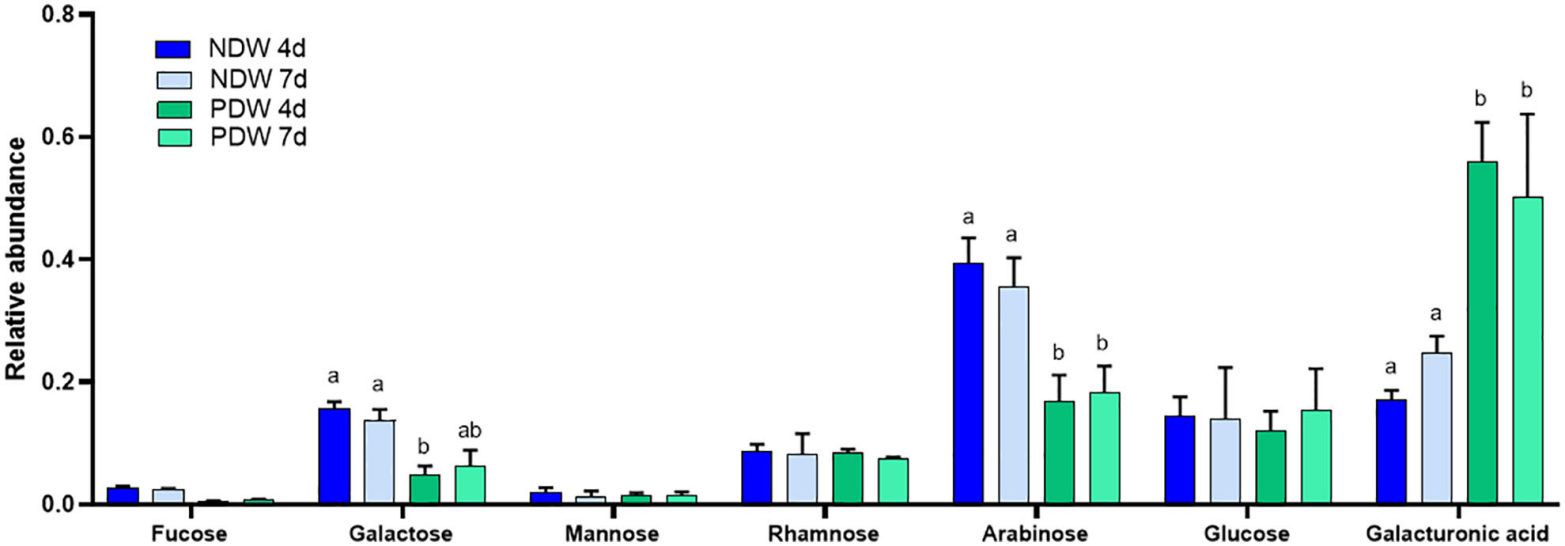
Monosaccharide analysis of NDW and PDW fractions. Histograms display the relative abundance of monosaccharides released upon AIR hydrolysis with 2M trifluoroacetic acid of NDW and PDW fractions obtained from 4- and 7-day-old *A.thaliana* cell cultures. Data are the mean of three biological replicates, error bars are the standard deviation. Lowercase letters indicate significant differences in the amounts of specific monosaccharides between fractions (two-way ANOVA, p < 0.05).

As the amount of PD material isolated is very low, PDW samples cannot be weighed and the results of enzymatic analyses have to be represented as relative data. Clear polysaccharide signals were nonetheless obtained from the monosaccharide analysis, indicating efficient retention of wall material in PDW fractions (**Figure 7**). This observation matches the preliminary TEM images (**Figures 2A** **–C**). A significant exception in detection was xylose, which we did not identify in any PDW sample (**Figure 7**). A low response factor or limited amounts of xyloses and xyloglucans, sources of this monosaccharide (Scheller and Ulvskov, 2010) might be the cause.

Clear differences between NDW and PDW fractions were also observed. Galacturonic acid, a sugar highly present in pectins (**Figure 3**; Voragen et al., 2009), was relatively more abundant in PDW (**Figure 7**). Galactan and arabinan monosaccharides, derived from side chains of RG-I (**Figure 3**; Zablackis et al., 1995) and/or arabinogalactan proteins (Ellis et al., 2010), instead displayed the opposite pattern (**Figure 7**). This might indicate low-substituted RG-I in PDW fractions.

**Figure 8:**
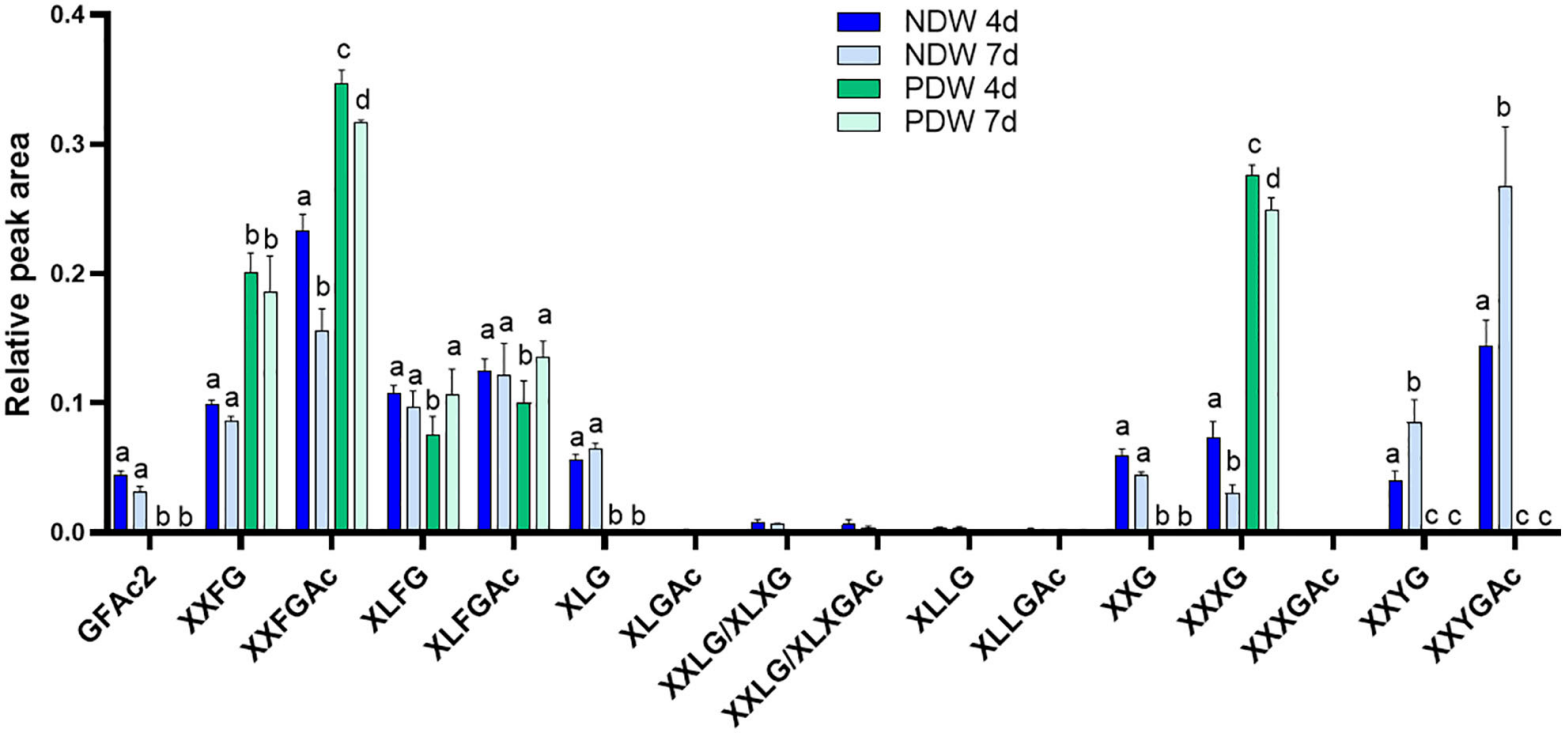
Enzymatic fingerprinting of xyloglucans in NDW and PDW fractions. Histograms display the relative peak area of each xyloglucan oligosaccharides released upon endo-cellulase digestion of NDW and PDW fractions obtained from 4- and 7-day-old *A. thaliana* cell cultures. Data are the mean of three biological replicates, error bars are the standard deviation. Lowercase letters indicate significant differences in the amounts of specific xyloglucans between fractions (two-way ANOVA, p < 0.05). Oligosaccharide nomenclature follows that described in Fry et al. (1993): G stands for glucose; X for xylose-glucose; L for galactose-xylose-glucose; F for fucose-galactose-xylose-glucose; Y for galacturonic acid-xylose-glucose; Ac: acetyl ester group.

Very few types of xyloglucans - mostly acetylated/non-acetylated-fucose-containing forms (XXFG and XLFG) - were detected in the PDW fraction (**Figure 8**). A loss of these species had indeed been observed from NDW fractions upon “Onozuka” R10 cellulase digestion (**Figure 4**). Galacturonic acid containing species, whose abundance was similarly reduced in DIW, were conversely not present in PDW. Loss of the latter might be attributable to interactions with cellulose (Park and Cosgrove, 2015) or by direct galacturonidase activity in the enzymatic mixture. Unlike NDW, galactosylated (L) but non-fucosylated (F) species were absent in PDW (**Figure 8**).

**Figure 9:**
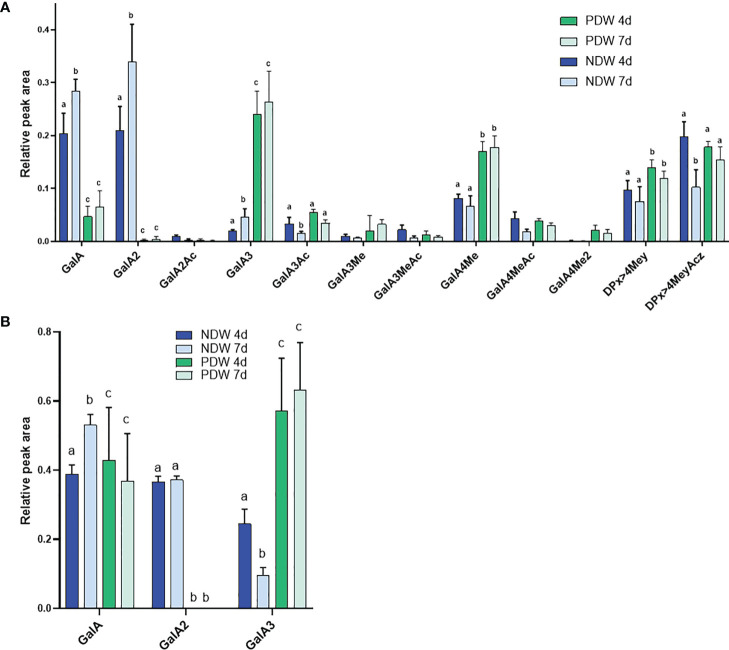
Enzymatic fingerprinting of HG in NDW and PDW fractions. Histograms display the peak area of each OGs analysed by HPSEC-HRMS and released upon endo-polygalacturonase digestion of non-saponified **(A)** and saponified **(B)** NDW and DIW fractions obtained from 4- and 7-day-old *A thaliana* cell cultures. Data are the mean of three biological replicates, error bars are the standard deviation. Lowercase letters indicate significant differences in the amounts of specific OGs between fractions (two-way ANOVA, p < 0.05). OGs are named GalAxMeyAcz. Numbers indicate the degree of polymerization and the number of methyl/acetyl ester groups. GalA, galacturonic acid; Me, methyl-ester group; Ac, acetyl-ester group; DP, Degree of polymerisation. OGs larger than GalA4 are grouped together. z and y represent potential higher levels of acetylation/methylation in those longer OGs.

Upon HG enzymatic fingerprinting, PDW fractions yielded significantly higher amounts of GalA3 and lower amounts of GalA compared to NDW. Surprisingly, no GalA2 was produced upon poly-galacturonase treatment (**Figure 9A**). Based on the association of GalA3 with non and/or lowly methyl-esterified wall regions and GalA/GalA2 with methyl-esterified ones, the HG in the PD environment might have low levels of substitution. Higher levels of galacturonic acid in the monosaccharide analysis (**Figure 7**) also suggest more pectins. Nonetheless, low methyl-esterified regions, of which the degradation leads to the release of GalA4Me, were clearly present in PDW fractions (**Figure 9A**). A full profile of OGs detected in PDW is provided in **Supplementary Figure S7**. As fewer OGs extend beyond GalA9Me4 in PDW compared to NDW, the methyl-esterified HG stretches at PD might be shorter.

Upon saponification, PDW fractions continued to release more GalA3 than NDW ones (**Figure 9B**) confirming that PD are enriched in HGs compared to NDW. Since GalA was detected in treated PDW fractions, the continued absence of GalA2 must be due to specific structural motifs such as the presence of side chains and/or of PG hydrolysable HG stretches amongst RG-I backbone rather than HG methylesterication. Large losses of methyl-esterified HG (GalA- and GalA2-associated) had been observed upon “Onozuka” R10 cellulase treatment in DIW fraction (**Figure 5B**). The PDW polysaccharide content does not seem to fully mirror this composition (**Figures 9A** **, B**).

**Figure 10:**
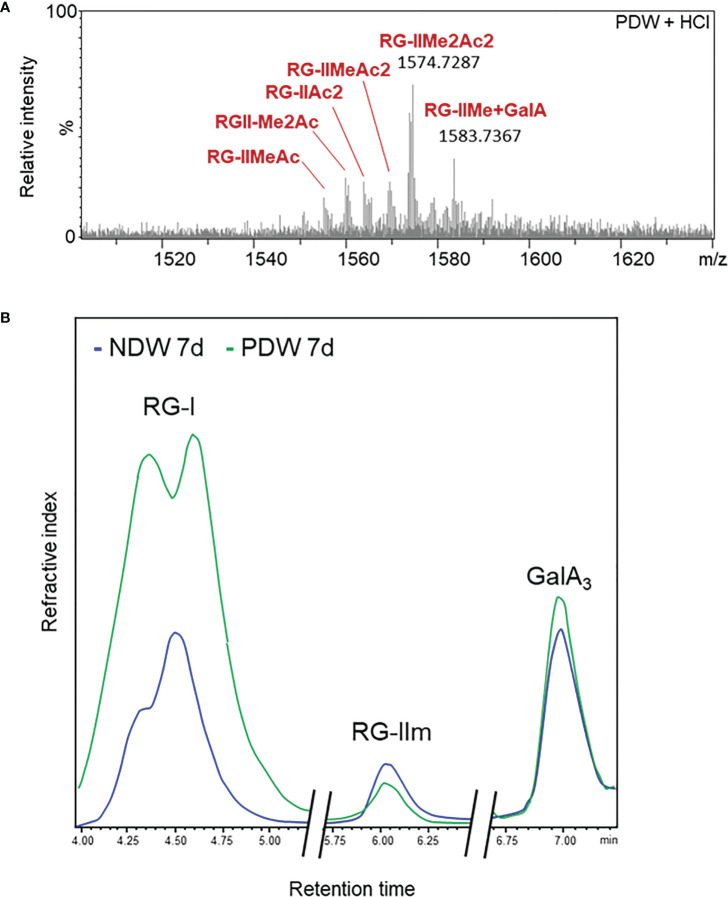
Rhamnogalacturonan characterisation of PDW. **(A)** Mass spectrum profile of complete monomerised RG-II forms in PDW fractions derived from 7-day-old *A.thaliana* cell cultures. Me and Ac denote methylation and acetylation in RG-II species. GalA; galacturonic acid. m/z: mass-to-charge ratio. **(B)** HPSEC-RI quantification of RG-I, RG-II monomers (RGIIm) and GalA3 in NDW and PDW fractions derived from 7-day-old *A.thaliana* cell cultures. The x axis is broken.

Due to the small amount of material in PDW fractions, it was not always possible to detect RG-II in those samples. A qualitative, rather than quantitative assessment, is therefore provided here. Upon HCl treatment, HPSEC-HRMS detected highly methyl-acetylated RG-II monomers, RG-IIMe_2_Ac_2_ being the main compound (**Figure 10A**). The degree of substitution was higher than that observed in NDW (**Figure 6A**). Loss of methyl-acetylated RG-II species had been observed upon “Onozuka” R10 cellulase digestion (**Figure 6B**), matching this PDW population.

The HPSEC-RI further supported the presence of RG-II monomers in PDW (**Figure 10B**). RG-II monomers and GalA3 were respectively slightly less and more abundant in PDW, compared to NDW (**Figure 10B**). The increased GalA3 abundance matches the previous OGs results (**Figures 9A** **, B**). HPSEC-RI also highlighted a high abundance of RG-I in PDW (**Figure 10B**), matching the loss observed in DIW (**Figure 6D**). A population of RG-I, corresponding to the first of the two peaks in the chromatogram, seems particularly prominent (**Figure 10A**).

Overall, the PDW fraction displayed a unique pattern of fucosylated xyloglucans; poorly methyl-esterified HGs with specific structures; potentially low-substituted RG-I species; and acetylated RG-II forms.

### PDW fractions displays limited age-related polysaccharide remodelling

As plant tissues mature, their cell walls (including those proximal to PD) can display morphological changes (Nicolas et al., 2017 as an example). To determine potential shifts in wall composition (involving xyloglucans or HGs), we compared fractions derived from 4- and 7-day-old cultures.

Monosaccharide abundances in PDW and NDW fractions did not seem to display temporal patterns. (**Supplementary Figure S3**, **Figure 7**), suggesting that the main classes of polysaccharide are stable in relative amounts. Limited remodelling of xyloglucans (mostly involving galactosylation - L species) was observed in PDW fractions between the two time points assessed (**Figure 8**). A more pronounced change in galacturonydation (Y species) was conversely detected in NDW xyloglucans (**Figure 4**).

Pectin-derived OGs were the polysaccharides displaying the most significant changes between 4- and 7-day-old cultures: digestible pectic patterns that lead to GalA and GalA2 production significantly increased within the interval (**Figure 5A**; **Supplementary Figure S4**). Concomitantly, a reduction in digestible pectic patterns leading to larger methyl-acetylated OGs (DPx>4MeyAcz) also occurred (**Figure 5A**; **Supplementary Figure S4**). Enzymes mediating *in muro* de-methyl-acetylation likely underpin these processes. Saponification, conversely, made apparent that the total HG content (from which GalA3 is produced after saponification) decreased with time (**Figure 5B**).

The PDW pectins from 4-day-old cultures were already highly de-methyl-esterified to start with. HG fingerprinting shows stable contents of the various OG classes between 4 and 7 days both before (**Figure 9A**) and after (**Figure 9B**) saponification. The only exception is GalA3Ac production which decreased in the time interval (**Supplementary Figure S7**).

Overall, compared to the broad cell wall, the PDW fraction seems to display more limited remodelling within the time window assessed.

## Discussion

In the last two decades, PD purification approaches have significantly increased our knowledge of lipids (Grison et al., 2015; Liu et al., 2020) and proteins (Fernandez-Calvino et al., 2011; Brault et al., 2019) closely associated with these structures. While most of the data has been obtained in cell culture systems, it effectively translates to intact plants (Brault et al., 2019). The third molecular component of PD, that of wall polysaccharides, had not yet benefited from the purification approach.

Here we provide a proof-of-concept attempt to address this aspect. We show that wall polysaccharides are co-purified in PD fractions (**Figures 2**, **7**–**10**) and that they can be effectively analysed with a combination of biochemical strategies (**Figure 3**). The polysaccharides in the PDW fractions most likely derive from the cell wall in the proximity of PD. Some of the observed signatures have indeed been independently reported by *in-situ* studies in the literature. However, we acknowledge the difficulty of inferring spatial proximity and *in-muro* composition from digestion products alone. Similar limitations might also apply to the absolute purity of the fractions: some PD (and associated polysaccharides) might not be released by the “Onozuka” R10 cellulase mixture and remain in DIW fractions. That said, their specific signatures would be highly diluted.

In this manuscript we focused in particular on xyloglucans and pectin components, which together account for around 60% of the plant cell wall (Zablackis et al., 1995). These components are also suitable for enzymatic fingerprinting, an approach with increased descriptive power and quantitative precision (Jermendi et al., 2022). These aspects make fingerprinting an ideal approach to study wall microenvironments. Polysaccharide microdomains - with the exception of xylem pits (Wang et al., 2022) - remain poorly defined to date.

In PDW fractions the failed detection of xylose might indicate a lower abundance of xyloglucans and xylans, sources of this monosaccharide (**Figure 7**). Immunolabeling studies in different plant species similarly reported that PD positions appeared to have low amounts of xyloglucans (Vaughn et al., 1996; Sutherland et al., 1999). Here we provide details on the composition of the xyloglucan species that are present, which happen to be highly fucosylated in the PDW fraction (**Figure 8**). Xyloglucans participate in interactions with cellulose (Park and Cosgrove, 2015) and fucosylated species seem to play a specific role in this interaction (Levy et al., 1991). The loss of galacturonic acid containing xyloglucans upon “Onozuka” R10 cellulase treatment, is also compatible with this notion (**Figure 4**, NDW versus DIW). Interestingly, the PDW fraction, despite experiencing the same cellulase treatment, retained fucosylated xyloglucan profiles. These forms might remain unaffected (or protected) at PD because they do not interact with cellulose (which is depleted around PD - Faulkner et al., 2008) or because they engage with other local wall polysaccharides. This is a relevant area of future work that could be addressed in a manner similar to Abou-Saleh et al., 2018. Differential abundance of fucosylated species might be ultimately biologically relevant. Accumulation of xylans/non-fucosylated xyloglucans at PD seemed to occur in compatible plant-pathogen interactions (Otulak-Kozieł et al., 2018). Lastly, a more diverse range of xyloglucan patterns was observed in the cell culture fractions compared to the cell wall of specialised tissues in plants. For example, in our NDW fractions we identified acidic forms containing galacturonic acid, xylose, and glucose (YXXG/YXXGAc), previously only reported in root hair cell walls (Peña et al., 2012) (**Figure 4**).

Enrichments in low-esterified HGs at PD have been described in multiple species using antibodies (Roy et al., 1997; Orfila and Knox, 2000; Faulkner et al., 2008; Giannoutsou et al., 2013). The high levels of GalA3 we quantified in PDW fractions (**Figure 9A**; **Supplementary Figure S7**) are in good agreement with this. Based on the comprehensive list of OGs released upon polygalacturonase action, we also show that methylation is present in short stretches (**Supplementary Figure S7**). This feature could be important to provide wall flexibility in accommodating PD aperture changes. Altering the level of pectin methylation at PD - *via* PME or PME inhibitor proteins - indeed influences plant-virus interactions (Lionetti et al., 2014b). In addition, highly de-methyl esterified HG could be indicative of a high pH in the PD wall environment. PMEs are indeed processive under those conditions (Hocq et al., 2021 - bioRxiv). A member of another group of pH dependent cell-wall modifying proteins, that of expansins, has also been localised to PD (Park et al., 2017).

The persisting lack of GalA2 release in the saponified PDW fractions (**Figure 5B**) could be linked to the existence of short HG stretches inside an RG-I backbone (Rha-GalA-GalA-GalA-GalA-Rha) (Yapo et al., 2007). The absence of such a domain in PDW would avoid the polygalacturonase to hydrolyze RG-I in smaller subdomains. Consistent with this assumption, we observed two populations of RG-I and PDW fractions are relatively enriched in the larger one (**Figure 10B**). The lower levels of arabinose and galactose monosaccharides in PDW, compared to the other fractions (**Figure 7**), do not mirror the reported abundance of arabinan-containing pectins and depletion of galactan-containing ones (Roy et al., 1997; Orfila and Knox, 2000; Faulkner et al., 2008). Gal/Ara ratios are actually roughly equal across all fractions (**Figure 7**; **Supplementary Figure S3**). Differences between tissues, species and the non-pectin-specific nature of the monosaccharide analysis could all be plausible explanations for this. Interestingly, RG-I has been shown to be an important determinant of cell-cell adhesion in wood cell walls (Yang et al., 2020). As PD might be viewed as mechanical weakness points in the otherwise solid cell wall, it is tantalising to speculate that the RG-I there might also carry similar functional roles.

The discrepancy between NDW-DIW and PDW composition in terms of OGs (**Figures 5**, **9**) and galacturonic-acid-containing xyloglucans (e.g. YXXG) (**Figures 4**, **8**) indicates that a fraction of HG and xyloglucans might have been directly digested by enzymatic activities in the mixture. Loss of HG could support our initial speculation that these polysaccharides act as the attachment sites that retain PD in the wall (**Figure 1**). Overall, in assessing the results presented in this paper, it is important to keep in mind that lack of detection of specific polysaccharides in DIW or PDW fractions does not imply absolute absence *in muro*. In the future, PD might be released from the wall with the use of more specific enzymes matching the local polysaccharide composition rather than with the “Onozuka” R10 cellulase treatment. We hope that the data in this manuscript will help the field move in such a direction.

The highly methyl-acetylated RG-II species detected at PD could suggest a non-random distribution of this polysaccharide in the plant cell wall (**Figure 10A**). This would be a novel concept. The lower extraction efficiency could also indicate reduced RG-II presence (in proportion to the small amount of PD material) or reflect tighter binding of these pectins in those fractions, causing inefficient extraction. RG-II have indeed been shown to directly bind GIPCs lipids (Voxeur and Fry, 2014), postulating interactions between the wall and the outer leaflet of the plasma membrane. Relevantly, those same lipid components are known to be enriched at PD (Grison et al., 2015; Liu et al., 2020). RG-II could act as structural elements that anchor PM-Wall and maintain the stability of PD in face of pressures favouring a collapse of PD membranes.

Our data additionally suggest that pectin and xyloglucan signatures linked to PD are present early on and experience limited changes, as the cell wall (and its PD) remodels (Nicolas et al., 2017) (**Figures 8**, **9**; **Supplementary Figure S7**). This is in contrast with the rest of the wall, which is more dynamic in methylation and acetylation modifications (**Figure 5**; **Supplementary Figure S4**). A unique PD wall environment might emerge already during phragmoplast and primary plasmodesmata formation, when numerous vesicles containing wall material are being delivered to the forming wall (Seguí-Simarro et al., 2004). Targeting of content-specific vesicles to determined positions (or exclusion from the same) would be required. An alternative explanation for the polysaccharide patterns would be that PD resident proteins alter the wall composition *in muro*, shortly after the establishment of primary PD (Hepler, 1982). This has been described in the context of callose (Levy et al., 2007; Vatén et al., 2011). Several pectin modifying proteins have also been detected in the PD proteome (Knox and Benitez-Alfonso, 2014).

Overall, we hope that the PD biology community will test and perfect this strategy to characterise PD wall polysaccharides. Applying it to a range of developmental, environmental and genetic situations will bring further robustness to association claims and might eventually unravel relevant aspects of cell-cell transport regulation and PD structural integrity.

## Methods

### PDW purification

PDW fractions were obtained from *A.thaliana* suspension cultured cells of the ecotype *Landsberg erecta*. Four- and seven-day-old cells (3 flasks of 200 mL each) were spun at 800 g for 5 min and resuspended with 25 ml of cold wall-preparation buffer (100 mM Tris-HCl, pH 8.0, 100 mM KCl, 10% v/v glycerol, 10 mM ethylenediaminetetraacetic acid, 0.45 M mannitol, and a complete protease inhibitor cocktail). The cells were passed four times through a N_2_ disruption bomb: the preparation was equilibrated for 5 min under N_2_ at 120 bars and then passed slowly through the release valve. After centrifugation at 400 g at 4°C for 5 min the pelleted walls were ground to a fine powder in liquid N_2_, then washed with cold wall-washing buffer (10 mM Tris-HCl, pH 8.0, 100 mM NaCl, 10% v/v glycerol, 10 mM ethylenediaminetetraacetic acid) by sequential centrifugations at 400 g for 5 min. The wall fraction obtained was named NDW.

These purified cell walls were then digested with 0.7% w/v cellulase “Onozuka” R10 (Karlan) in digestion buffer (10 mM MES, pH 5.5, and 4.4% mannitol) containing 1 mM phenylmethylsulfonyl fluoride and complete protease inhibitor cocktail for 1h30 h at 37°C with 200 rpm shaking. After centrifugation (5850 g) for 5 min at 4°C, the supernatant and pellet fractions were collected separately. The supernatant, which contain PD-structures, was process through ultracentrifugation at 110,000 g for 40 min at 4°C. The resulting pellet, called PDW, was washed in an excess volume of cold Tris-buffered saline (TBS; 20 mM Tris-HCl, 0.14 M NaCl, and 2.5 mM KCl, pH 7.4), spinned down again at at 110,000 g for 40 min at 4°C. Finally, the pellet was resuspended in cold 1X TBS containing protease inhibitors. Approximately 600 mL of cultured cells was used to obtain 200 µg protein-equivalent of PDW. Protein amount was determined with a bicinchoninic acid protein assay using BSA as standard.

### TEM microscopy

For immunogold labelling of the PDW fraction, 5µl of purified membranes, at a protein concentration of 0.1 mg/mL, were pipetted onto plastic- and carbon-coated grids. Excess liquid was removed, and the grids were incubated with 10 mL of PBS blocking buffer (0.15 M NaCl, 7.5 mM Na_2_HPO_4_, and 0.25 mM NaH_2_PO_4_ containing 5% bovine serum albumin, 5% normal goat serum, and 0.1% cold water fish skin) for 1 h and then washed with 1X PBS containing 0.05% Tween 20, before being incubated for 90 min with the primary antibodies diluted in PBS 0.1% acetylated BSA. Dilutions of 1:100, 1:30, and 1:30 were used for PDCB1 (Simpson et al., 2009), PDLP1 (Thomas et al., 2008), and callose (Biosupplies monoclonal antibody) antisera. Controls were performed with pre-immune and second antibodies only. After six washes (5 min each with PBS 0.05% Tween 20) antibody binding was detected using 5 nm and 10 nm gold-conjugated goat anti-rabbit and anti-mouse antibodies diluted at 1:30. After 1 h of incubation at room temperature, the grids were washed six times with PBS 0.05% Tween 20 and three times with 0.2 μm filtered water and then negatively stained with 2% (w/v) uranyl acetate. PDW and DIW observations were carried out on a FEI TECNAI Spirit 120 kV electron microscope.

### Cell wall monosaccharide composition

NDW, DIW and PDW fractions were submerged in 96% (v/v) ethanol and boiled at 70°C for 10 min. The pellets were collected by centrifugation (13000 g for 10 min) and dried in a speed vacuum concentrator at 30°C overnight. The monosaccharide compositions of the non-cellulosic fractions were determined by hydrolysis of 100 µg AIR with 2 M trifluoroacetic acid for 1 h at 120°C. After cooling and centrifugation, the supernatant was dried under a vacuum, resuspended in 200 µL of water and analysed by high-performance anion-exchange chromatography/pulsed amperometric detection on a Dionex ICS5000 instrument (ThermoFisher Scientific) as described in Fang et al., 2016.

### Enzymatic fingerprinting of pectins and xyloglucans

The PDW, DIW and NDW fractions were dried in a speed vacuum concentrator at 30°C overnight. Samples were digested with 1 U/mg DW sample of *Aspergillus aculeatus* endo-polygalacturonase M2 or endo-cellulase (Megazyme, Bray, Ireland) (Lerouxel et al., 2002) in 50 mM ammonium acetate buffer (pH 5) at 37°C for 18 h. Samples were then centrifuged at 13000 rpm for 10 min and 100 µL of the supernatants were transferred into vials. For MS analysis, 10 μl of each fraction were injected in the machine.

### Oligosaccharide characterization and quantification by LC/HRMS analysis

The oligosaccharides released from digestion were separated according to Voxeur et al., 2019. Chromatographic separation was performed on an ACQUITY UPLC Protein BEH SEC Column (125A, 1.7 μm, 4.6 mm X 300 mm, Waters Corporation, Milford, MA, USA) coupled with guard Column BEH SEC Column (125A, 1.7 μm, 4.6 mm X 30 mm). Elution was performed in 50 mM ammonium formate, 0.1% formic acid at a flow rate of 0.4 mL/min and with a column oven temperature of 40°C. The injection volume was set to 10 μl. The quantitative evaluation of xyloglucan fragments was made using an HPLC system (UltiMate 3000 RS HPLC system, Thermo Scientific, Waltham, MA, USA). The system was coupled to an Impact II Ultra-High Resolution Qq-Time-Of-Flight (UHR-QqTOF) spectrometer (Bruker Daltonics, Bremen, Germany) equipped with an electrospray ionisation (ESI) source in negative mode with the end plate offset set voltage to 500 V, capillary voltage to 4000 V, nebulizer to 40 psi, dry gas to 8 l/min and dry temperature of 180°C. The Compass 1.8 software (Bruker Daltonics) was used to acquire the data.

The Mzmine 2.53 software was used to analyse data according to Pluskal et al., 2010. To perform integration, the filter noise level for mass detection was set to 500. The ADAP Chromatogram Builder (Myers et al., 2017) was used with the following parameters: range of 6 - 10 min; min group size of scan 10; group intensity threshold of 1000; min highest peak of 500;and m/z tolerance of 0.01 or 10 ppm. The chromatogram peaks were deconvoluted using aBaseline cut-off of 300. Chromatograms were manually de-isotoped and peaks aligned (m/z tolerance of 0.01 or 5 ppm; retention time tolerance of 0.1).

## Data availability statement

The raw data supporting the conclusions of this article will be made available by the authors, without undue reservation.

## Author contributions

EMB and AV designed the experiments. AP wrote the manuscript with input from all other authors. AP, FI, MSG extracted wall and PD fractions from cell cultures. EMB performed TEM microscopy. JS and GM performed monosaccharide analysis. SP performed mass spectrometry analysis. AV performed all other analyses. All authors contributed to the article and approved the submitted version.

## Funding

This work was supported by the European Research Council (ERC) under the European Union’s Horizon 2020 research and innovation programme (Grant agreement No 772103-BRIDGING) to E.M.B; the EMBO Young Investigator Program to E.M.B; the AAP INNOVATION et ENTREPRENARIAT- PREMATURATION 2018 to A.V (Grant agreement No CDE-2018-002330-IRE 2018-0024, OGome). This work also benefited from IJPB’s Plant Observatory technological platforms, which are supported by Saclay Plant Sciences-SPS (ANR-17-EUR-0007).

## Conflict of interest

The authors declare that the research was conducted in the absence of any commercial or financial relationships that could be construed as a potential conflict of interest.

## Publisher’s note

All claims expressed in this article are solely those of the authors and do not necessarily represent those of their affiliated organizations, or those of the publisher, the editors and the reviewers. Any product that may be evaluated in this article, or claim that may be made by its manufacturer, is not guaranteed or endorsed by the publisher.
