# Supplementary material for: Enzymatic fingerprinting reveals specific xyloglucan and pectin signatures in the cell wall purified with primary plasmodesmata
Source: Front Plant Sci. 2022 Oct 25;13:1020506. doi: 10.3389/fpls.2022.1020506 (PMC9640925; doi:10.3389/fpls.2022.1020506)
Supplement: Supplementary file 1 [file DataSheet_1.pdf]

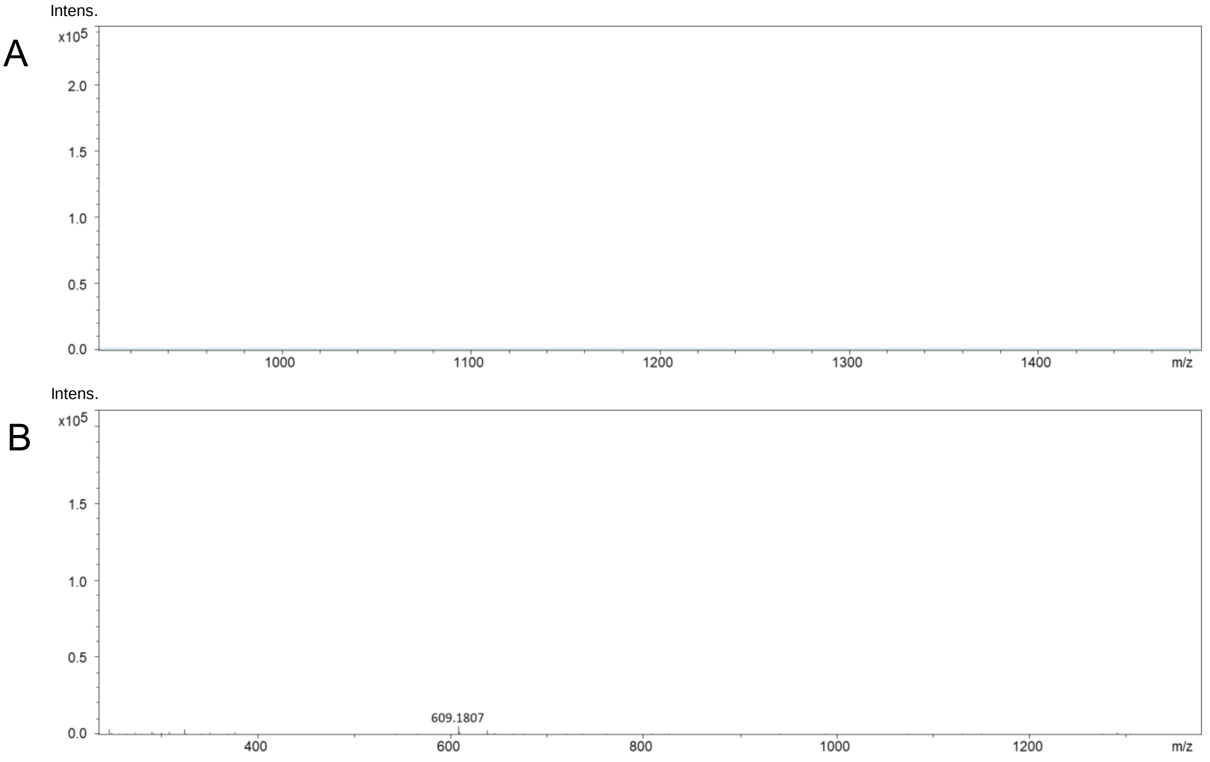

Supp. Fig. 1. Control MS spectra for “Onozuka” R 10 cellulase treatment. MS spectra obtained from untreated tamarind xyloglucan (**a**) and citrus pectins (**b**). The scale used is similar to Fig. 1c and d to allow direct comparisons. Intens: signal intensity; m/z: mass to charge ratio.

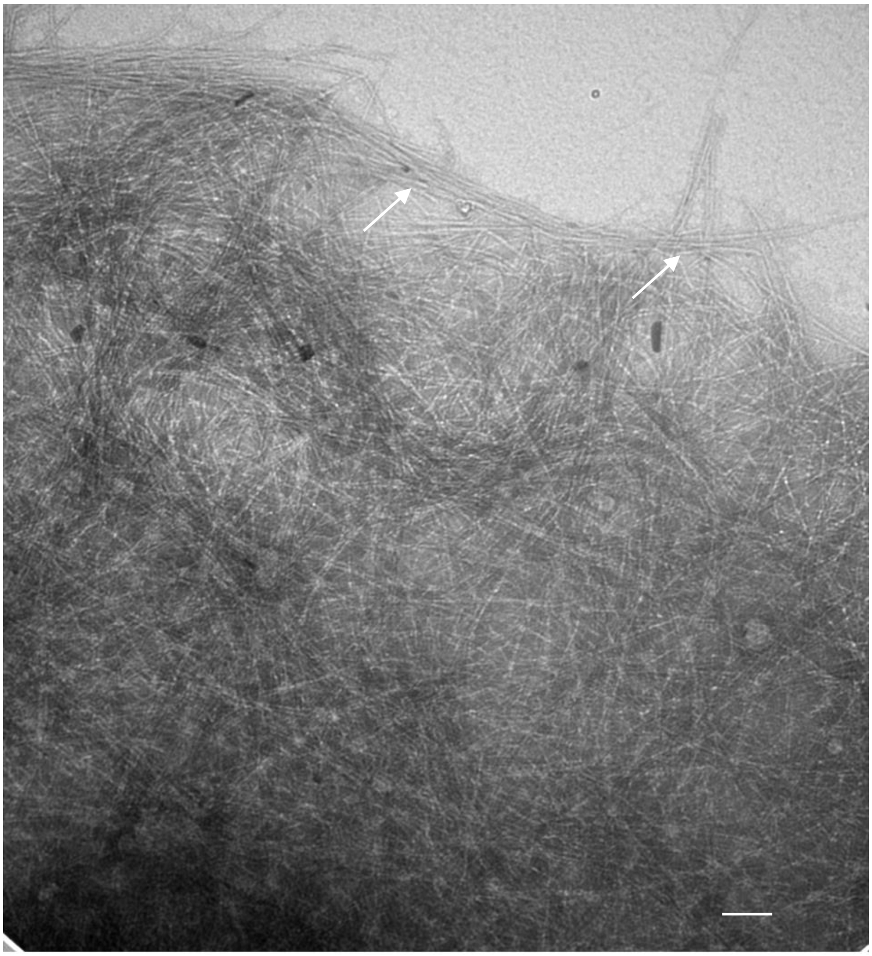

Supp. Fig. 2. Wall-derived material present in DIW fractions purified from *A.thaliana* cell cultures. TEM images of DIW fractions. Fibrillar structures are visible (white arrows). Scale bars of 100 nm are included.

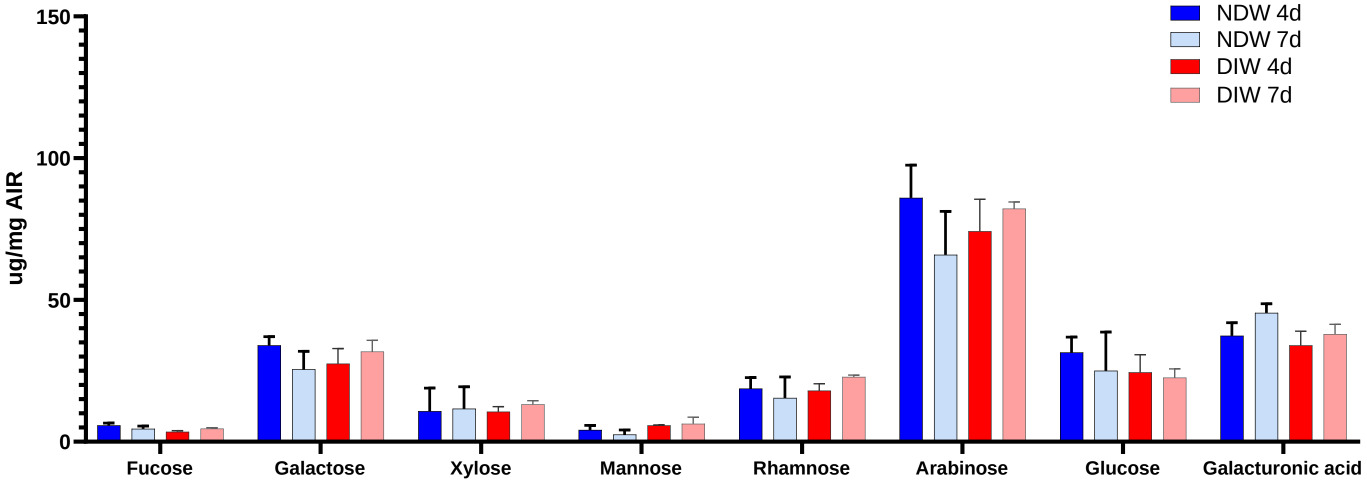

Supp. Fig. 3. Monosaccharide analysis of NDW and DIW fractions derived from 4 and 7-day-old *A.thaliana* cell cultures.

Histograms display the amount of monosaccharides released upon alcohol-insoluble residue hydrolysis with 2 M trifluoroacetic acid and analyzed by high-performance anion exchange chromatography with pulsed amperometric detection. Data are the mean of three biological replicates, error bars are the standard deviation

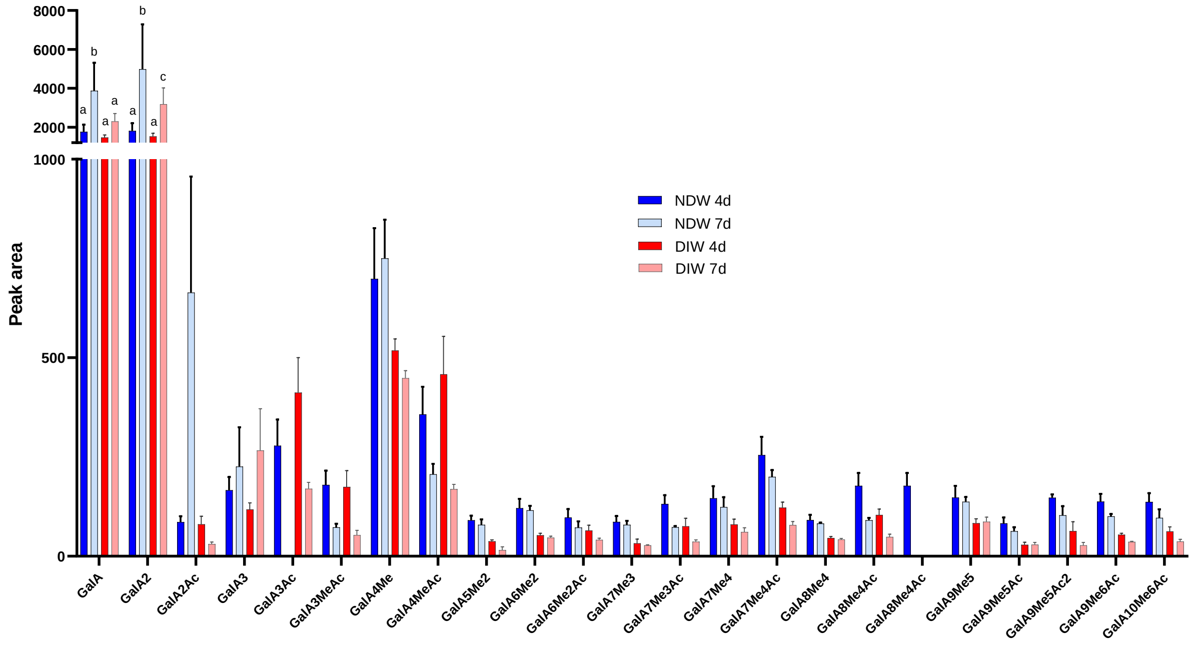

Supp. Fig. 4. Full profile of oligogalacturonides analysed by HPSEC-HRMS and produced upon PG digestion of NDW and DIW fractions. Histograms display the peak area of each oligogalacturonides released upon endo-polygalacturonase digestion of NDW and DIW fractions obtained from 4- and 7-day-old *A.thaliana* cell culture. Data are the mean of three biological replicates, error bars are the standard deviation. Lowercase letters indicate significant differences in the amounts of specific oligogalacturonides between fractions (two-way ANOVA, $p < 0.05$). OGs are named GalAxMeyAcz. Numbers indicate the degree of polymerization and the number of methyl and acetyl ester groups. GalA: galacturonic acid; Me: methylester group; Ac: acetylester group. The y axis is broken with a change in scale.

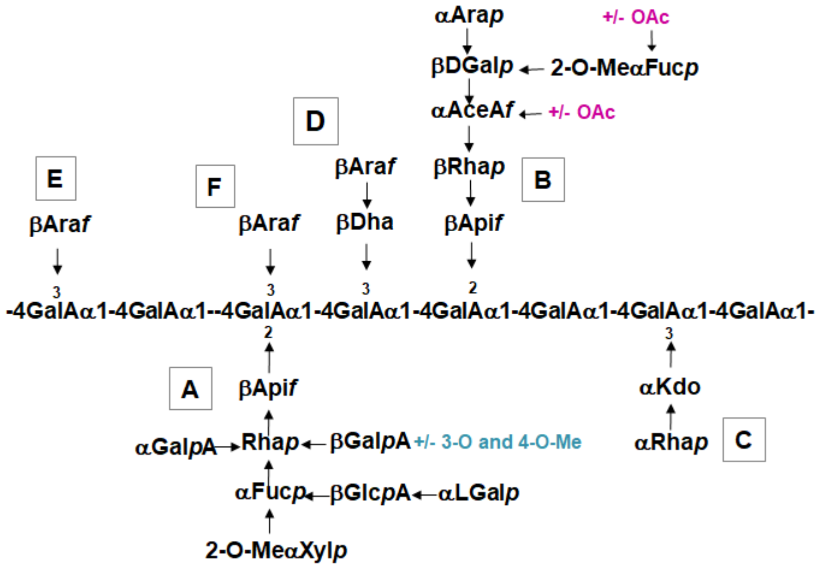

Supp. Fig. 5. Structure of RG-II pectin. Capital letters indicate the side chains along the RG-II backbone. Sugars: Gal (galactose); Fuc (fucose); Ara (arabinose); AceA (aceric acid); GalA (galacturonic acid); Glc (glucuronic acid); Api (apiose); Rha (rhamnose); Dha (3-deoxy-D-*lyxo*-2-heptulosaric acid); Kdo (3-deoxy-D-*manno*-2-octulosonic acid); Xyl (xylose). A lowercase terminal *p* indicates a pyranose sugar, *f* a furanose one. Initial *α* (alpha) or β (beta) symbols refer to the configuration of the anomeric carbon. A capital D or L indicates dextro/levo-rotatory sugar enantiomers. *O*-Ac and *O*-Me denote methyl and acetyl groups bound to an oxygen atom in the sugar. Numbers refer to the linkage between sugars/groups.

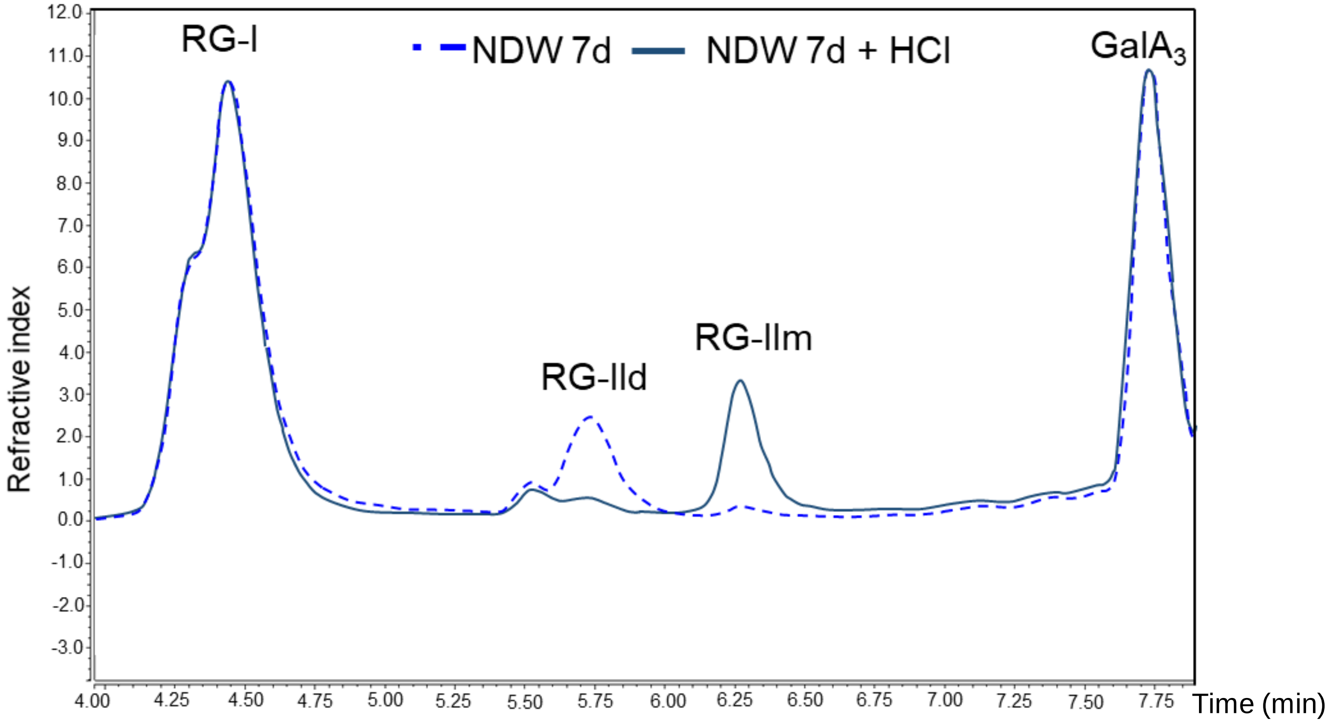

Supp. Fig. 6. HPSEC-RI detection of pectic components obtained following digestion of saponified NDW fractions derived from 7-day-old *A.thaliana* cell cultures before and after HCl treatment. The dashed line represents the untreated NDW fraction while the full line depicts the HCl treated one. Trimers of galacturonic acid (GalA3), Rhamnogalacturonan I (RG-I), Rhamnogalacturonan II monomers (RG-IIm) and dimers (RG-IId) are displayed.

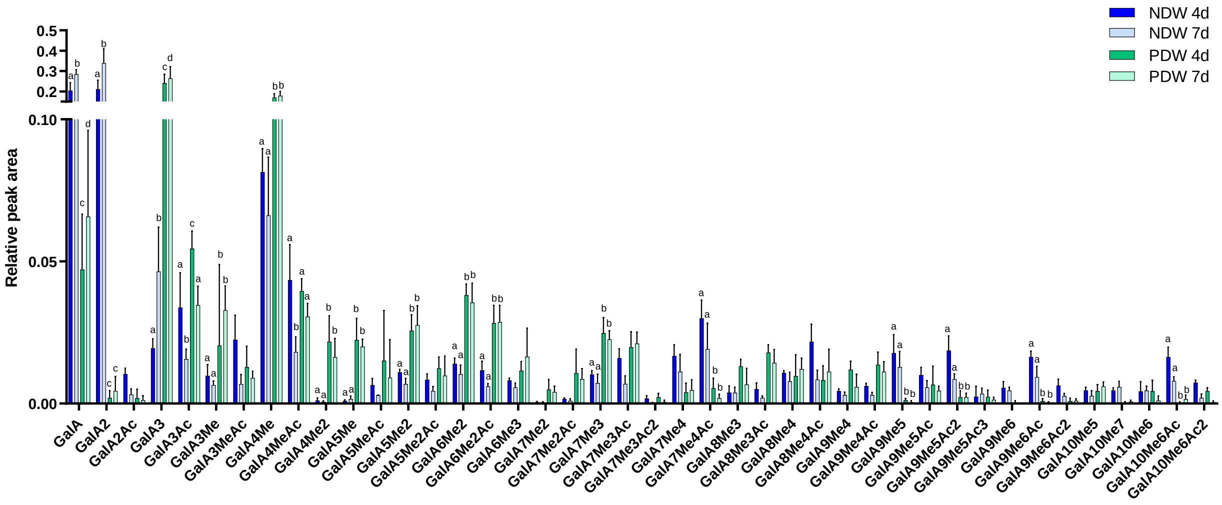

Supp. Fig. 7. Full profile of OGs produced upon PG digestion of NDW and PDW fractions. Histograms display the peak area of each oligogalacturonides analysed by HPSEC-HRMS and released upon endo-polygalacturonase digestion of NDW and PDW fractions obtained from 4- and 7-day-old *A. thaliana* cell culture. Data are the mean of three biological replicates, error bars are the standard deviation. Lowercase letters indicate significant differences in the amounts of specific oligogalacturonides between fractions (two-way ANOVA, $p < 0.05$). OGs are named GalAxMeyAcz. Numbers indicate the degree of polymerization and the number of methyl ester groups. GalA: galacturonic acid; Me: methylester group; Ac: acetylester group. The y axis is broken with a change in scale.
